# Sterile protection against relapsing malaria with a single-shot vaccine

**DOI:** 10.1038/s41541-022-00555-0

**Published:** 2022-10-27

**Authors:** Erica M. Pasini, Annemarie Voorberg van der Wel, Nicole Heijmans, Onny Klop, Anne-Marie Zeeman, Herman Oostermeijer, Ivonne Nieuwenhuis, Roberto Rodriguez Garcia, Nicole Onur van der Werff, Sam O. Hofman, Frank A. W. Verreck, Edmond J. Remarque, Bart W. Faber, Clemens H. M. Kocken

**Affiliations:** 1 Department of Parasitology, Biomedical Primate Research Centre, Rijswijk, The Netherlands; 2 Department of Virology, Biomedical Primate Research Centre, Rijswijk, The Netherlands

**Keywords:** Malaria, Cell vaccines

## Abstract

Vaccine development for *Plasmodium vivax*, an important human relapsing malaria, is lagging behind. In the case of the most deadly human malaria *P. falciparum*, unprecedented high levels of protection have been obtained by immunization with live sporozoites under accompanying chemoprophylaxis, which prevents the onset of blood-stage malaria. Such an approach has not been fully evaluated for relapsing malaria. Here, in the *P. cynomolgi*-rhesus macaque model for relapsing malaria, we employ the parasites’ natural relapsing phenotype to self-boost the immune response against liver-stage parasites, following a single-shot high-dose live sporozoite vaccination. This approach resulted in sterile protection against homologous sporozoite challenge in three out of four animals in the group that was also exposed for several days to blood stages during primary infection and relapses. One out of four animals in the group that received continuous chemoprophylaxis to abort blood-stage exposure was also protected from sporozoite challenge. Although obtained in a small number of animals as part of a Proof-of-Concept study, these results suggest that limited blood-stage parasite exposure may augment protection in this model. We anticipate our data are a starting point for further research into correlates of protection and extrapolation of the single-shot approach to develop efficacious malaria vaccines against relapsing human malaria.

## Introduction

**Fig. 1:**
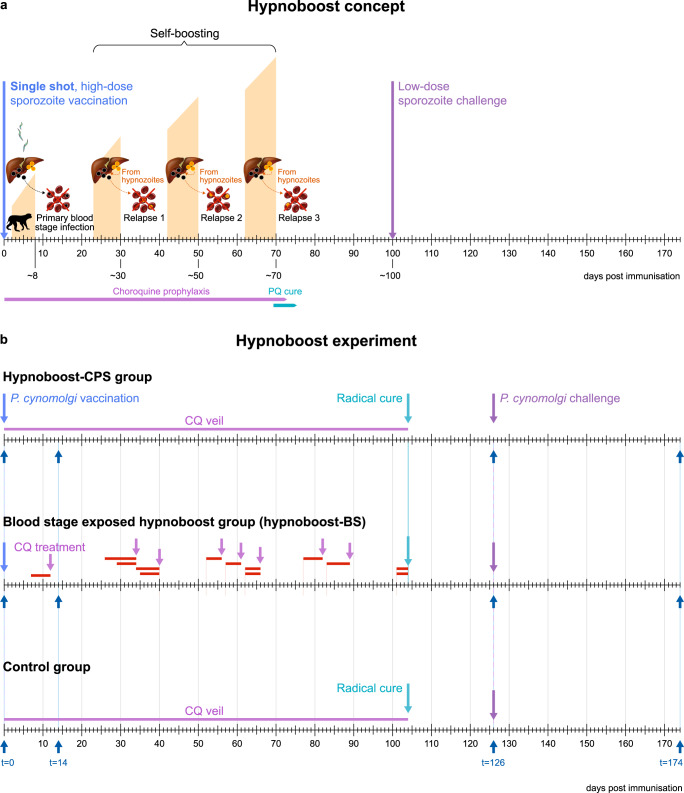
Hypnoboost concept and experimental set-up. **a** The hypnoboost concept consists of a single-shot high-dose relapsing malaria sporozoite vaccination to induce and boost liver-stage immune responses (orange background bars), while aborting blood-stage infection by continuous chloroquine treatment. Following liver-stage parasite clearance (primaquine (PQ) treatment) after three relapses, immunity should be high enough to protect from sporozoite challenge. **b** The hypnoboost concept was tested using three groups of rhesus macaques (*n* = 4): a hypnoboost-CPS group, a blood-stage exposed hypnoboost-BS group and a control group. Light blue arrows: vaccination with 10^6^ *P. cynomolgi* sporozoites. Lila lines: chloroquine (CQ) coverage established by weekly CQ injections. Lila arrows: start of 5-day CQ treatment. Red lines: duration of blood-stage parasitemia in the four animals of the hypnoboost-BS group. Teal arrows: start of radical cure treatment (5-day CQ and 7-day primaquine). Purple arrows: challenge with 200 *P. cynomolgi* sporozoites. Dark blue arrows: bleeding time points.

Malaria continues to represent a serious health concern worldwide. In the Asia-Pacific and the Americas, a shift in malaria species composition has been observed, with *P. vivax* representing 53% of the global burden in South-East Asia and being the predominant species in the Americas^1^. This shift towards relapsing malaria affects both malaria control and eradication attempts. Efforts to tackle this challenge rely on both a better understanding of protective immunity to malaria and the translation of this knowledge into new, more effective vaccines against the parasite^2^. High levels of protection have been obtained by immunization with live sporozoites under accompanying chemoprophylaxis (chemoprophylaxis and sporozoite, CPS approach^3^), which prevents the onset of blood-stage malaria. In the CPS approach, human volunteers are exposed at least three times too low levels of infectious *P. falciparum* malaria sporozoites to initiate hepatocyte infection and subsequent symptomless full liver-stage development. Continuous chloroquine coverage is provided to abort blood stages, that follow upon completion of liver-stage development and are responsible for malaria pathology. This protocol mounts sufficient immunity to liver stages to provide solid protection from sporozoite challenge, possibly by the absence of prolonged blood-stage exposure^3–7^. Here, we followed a modified concept of the successful malaria sporozoites under a continuous blood-stage prophylaxis approach (CPS^3^). We present an innovative approach to a live sporozoite malaria vaccine for relapsing malaria with a single-shot, self-boosting vaccine (Fig. 1a), where naturally occurring relapses boost liver-stage immunity originating from the primary liver-stage development. Such single-shot approach would have enormous logistical benefits over vaccines that require regular booster injections when rolled out in countries with restricted access to health care. In this study, the long-standing gold standard experimental model for relapsing malaria (reviewed in ref. ^2^) was used: *P. cynomolgi* sporozoite-infected rhesus monkeys. *P. cynomolgi* is genetically very similar to *P. vivax*^8^ and considered the *P. vivax* sister parasite (reviewed in ref. ^2^). In the rhesus monkey, it displays near identical biology to *P. vivax*, including the frequent relapsing phenotype due to reactivating dormant liver-stages (hypnozoites)^2,9^. Importantly, it naturally infects rhesus macaques^9^ and humans^10,11^, and thus provides a natural model in a spleen-intact non-human primate host to study relapsing malaria vaccine immunogenicity, safety and efficacy. Rhesus monkeys are closely related to humans and have a similar immune system, making this model ideally suited for investigating and providing proof of concept of novel prophylactic strategies for possible translation to clinical evaluation^12^.

## Results

### Sterile protection in 75% of the animals following single-shot vaccination and limited blood-stage exposure

**Fig. 2:**
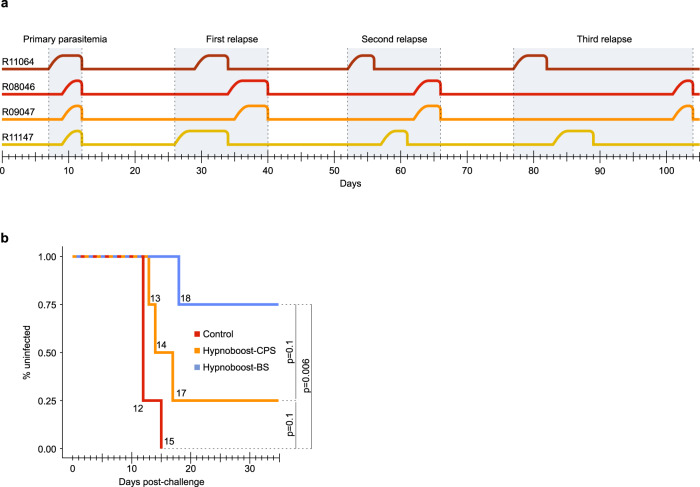
Relapse patterns and challenge outcome. **a** Primary parasitemia and relapse patterns of the four monkeys in the hypnoboost-BS group as monitored by daily blood smears, in days post vaccination. **b** Day to patency post *P. cynomolgi* sporozoite challenge of animals in the control group (red line), the hypnoboost-CPS group (orange line) and the hypnoboost-BS group (blue line). In total four of eight vaccinated animals were sterily protected. *p*-values are given for the inter-group comparison computed using R (survival package; log-rank test).

Our Proof-of-Concept study included three groups of four rhesus macaques: a CPS-like group that received one intravenous vaccination dose of 10^6 ^*P. cynomolgi* M-strain infectious sporozoites followed by a continuous choloroquine coverage (hypnoboost-CPS group); a blood-stage-exposed hypnoboost group (hypnoboost-BS, to monitor the effect of limited blood-stage exposure on liver-stage immunity), where vaccination was followed by chloroquine treatment several days post-blood-stage onset at primary infection and each of three relapses; and a mock-vaccinated control group (Fig. 1b). This set-up with a limited number of animals was chosen out of ethical reasons as there was no way of knowing beforehand if this approach would work given that there was a possibility that too few hypnozoites would relapse at any given time to yield a boost of the initial immune response. Since no data exist on how many hypnozoites relapse at any given time, we arbitrarily allowed three successive relapses on top of the primary liver-stage development to boost liver-stage immunity. Blood-stage parasites cannot be detected by thin blood-smear analysis in the hypnoboost group due to the chloroquine coverage. Therefore, the hypnoboost-BS group was used to monitor the primary blood-stage parasitemia and relapses. As expected, all four animals in the hypnoboost-BS group developed primary parasitemia between days seven and 11, and a total of three relapses in the hypnoboost-BS group was evident by day 101 post sporozoite injection (Fig. 2a; Supplementary data**:** Table 1). Blood-stage parasitemias were allowed for at least four days at each appearance before chloroquine treatment (Supplementary data**:** Table 1), to be able to monitor the effect of blood-stage exposure on the development of liver-stage immunity, which has been suggested to have a negative effect^3,4,13–15^. We stopped the vaccination phase in all animals after the third relapse in the last animal of the hypnoboost-BS group was evident (Fig. 1b, Fig. 2a). At that time, all twelve experimental animals, including the four controls, were treated with a combination of primaquine and chloroquine, to eliminate developing and dormant liver-stages as well as possible blood-stage parasites in the two hypnoboost groups (Fig. 1b). Animals were allowed to recover for a period of 15 days to allow for complete drug clearance^16,17^ before all animals were challenged with 200 freshly isolated *P. cynomolgi* sporozoites. From day 8 after the challenge, the animals were monitored daily for parasitemia through thin film blood-smears.

Three control group animals became thin film positive on day 12 post-challenge, and the fourth animal on day 15 (Fig. 2b**;** Supplementary Fig. 1). In the hypnoboost-CPS group, three animals became positive on day 13, 14 and 17, respectively, while the fourth animal remained negative during 43 days of follow-up. Unexpectedly, in the hypnoboost-BS group, three animals remained negative for the follow-up period of 43 days post-challenge, while only one animal became blood-smear positive on day 18 (Supplementary data**:** table 2). Overall, four out of eight hypnoboost-vaccinated animals were completely protected during the follow-up period, and the best protection (three out of four animals) was observed in the group that was exposed to blood-stage parasites during the vaccination phase.

### Evaluation of the immune response

**Fig. 3:**
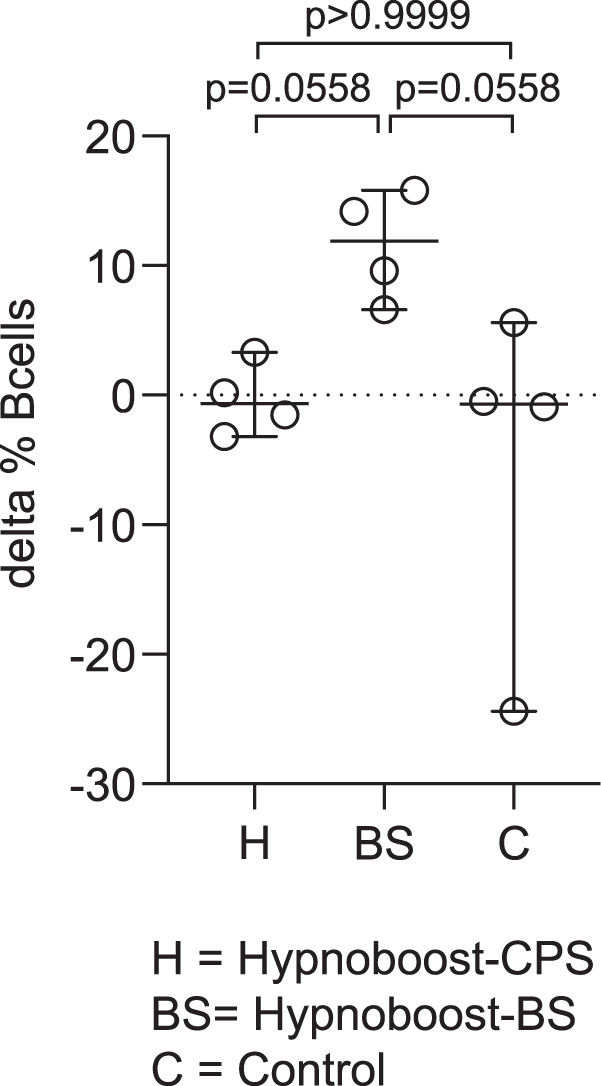
Difference between the B-cell frequency at t = 0 and just before challenge for the different groups. The B-cell compartiment (CD20 + HDLA + ) was analysed using flow cytometry. Results of the B-cell analysis for the individual animals in the hypnoboost-CPS (H), the hypnoboost-BS (BS) and the control (C) groups are shown as difference between the relative abundance of B cells just before t-challenge and at t = 0 (while the mean B-cell abundance was 18.3% on an average of 20,000 events analysed). Horizontal lines represent group averages. Next to the median, 95% CI are also indicated. *P*-values (by Kruskal–Wallis) are shown.

The limited exposure to blood stages in the hypnoboost-BS group was not sufficient to find antibodies by ELISA against selected blood-stage antigens (AMA1, MSP1, RON4, RON5, HSP70 and EXP1) nor by immunofluorescence on infected blood-smears. To assess the possible impact on the peripheral immune compartment of the experimental prophylactic immunisation with sporozoites in the presence or absence of chloroquine coverage, we monitored the frequencies of different immune cell subsets in the blood by spectral flow cytometry. To this end, we used frozen PBMC collected prior to treatment and 18 weeks later, prior to the infectious challenge. With the exception of CD20 + B lymphocytes, we found no apparent shifts in the frequencies of T lymphocytes, either CD4 + or CD8 + or gamma-delta-TcR + , MHC class-II-positive antigen-presenting cells (APC), including classical and intermediate monocytes and DC, or innate lymphocyte/NK cell populations that could be associated with the respective experimental treatment. Although statistical power was limited with only four animals per arm, the hypnoboost-BS group exclusively showed a trend of increased B-cell frequencies as a sign of exposure to antibody-inducing blood stages by the time of infectious challenge (Fig. 3).

## Discussion

The limited data available on induction of *P. vivax* liver-stage immunity stem from a study in Aotus monkeys with radiation-attenuated *P. vivax* sporozoite vaccination, where 10 subsequent vaccinations yielded 50% protection from sporozoite challenge^18^. In addition, a single sporozoite infection by two-four *P. cynomolgi*-infected mosquito bites in toque monkeys, while eliminating detectable blood stages monitored by daily blood smears and preventing relapses by primaquine treatment, showed signs of developing immunity to homologous sporozoite challenge^19^. The data we provide here with the hypnoboost concept demonstrates that solid protection against sporozoite challenge can be obtained in overall 50% of all eight vaccinated animals using relapsing malaria in a single-shot vaccination procedure. We used a high number of sporozoites (10^6^) in the immunization to increase the chance that sufficient numbers of hypnozoites would relapse at any given time to boost immunity. Such a high vaccination dose would not be practicable when translated to human trials. However, future experiments are needed to determine the lowest protective immunization dose, which may even be further decreased by the addition of appropriate adjuvants. One hypothesis on how CPS vaccines for *P. falciparum* work is that prevention of subsequent blood-stage infection is critical to inducing sterile protection by keeping the immune system focused on the liver stages^3,6^. Here we show, in a limited number of animals, that limited exposure to blood-stage parasites following full liver-stage development, may actually enhance the protective efficacy of this vaccine approach for *P. vivax*-type parasites (75% sterile protection in the hypnoboost-BS group). Our preliminary flow cytometric analysis of several immune cell subsets (T cells, B cells, monocytes, DC, innate lymphocytes/NK cells) revealed no salient differences between the protected and unprotected animals. More studies in this model are needed to address antigen-specific and/or tissue-resident immune cell responses (particularly of those that reside in the liver) to possibly identify immune correlates of protection and to further underpin the role of exposure to blood stages in developing protective immunity. In future, scRNAseq or other advanced cellular immunology tools will be applied to achieve a more in-depth investigation. Such investigation should also focus on the possibility that protected animals may have developed improved antigen-specific CD8 T cell responses from the boosting experiences. This would be particularly interesting in the context of a recent study that showed that CD8 T cells are able to recognize and kill *P. vivax*-infected reticulocytes, which bear MHC Class I^20^.

Recently, it was shown in humans that CPS vaccination using cryopreserved *P. falciparum* NF54 sporozoites results in sterile protection against challenge with the highly divergent, heterologous *P. falciparum* strain 7G8 in 10 out of 13 human volunteers^21^. It is not known whether cross-strain protection can be extended to cross-species protection in the case of closely related parasite species. However, it has been shown that *P. berghei* yields heterologous cellular immunity to *P. falciparum*^22^, and *P. cynomolgi* and *P. vivax* are closer related (sister parasites) than *P. falciparum* and *P. berghei*. While cross-species protection has still to be established in the model presented here, based on the current evidence, *P. cynomolgi*, a zoonotic parasite^11^ of the *vivax* clade, could possibly be developed into a cross-species vaccine for *P. vivax* and/or zoonotic *P. knowlesi* infection, given that a *P. vivax* sporozoite vaccine would be cumbersome to produce. In this context, further increasing the immunogenicity of the hypnoboost concept, e.g. by adding glycolipid-based adjuvants^23^, and/or adding relevant liver-stage antigens from *P. vivax/P. knowlesi*, could possibly increase the protective feature of hypnoboost in general and to other malaria species, without exposure to blood stages. The first step to take in this direction, following optimization of the homologous hypnoboost concept, is to investigate cross-species challenge with *P. knowlesi* in the non-human primate model. Subsequently, given that *P. cynomolgi* is amenable to genetic modification^24^, the engineering of parasites could be pursued: (1) to stop their development at the late liver-stage (Genetically Attenuated Parasites, GAP^25^), avoiding the need for chemoprophylaxis during vaccination, and (2) to express heterologous antigens. Such GAP parasites would continue relapsing in the liver; the booster component of the vaccination strategy thus remains intact. A single-shot self-boosting vaccine would have enormous (logistical) benefits for resource-poor settings over vaccines that need regular boosting. In addition, live sporozoite vaccine approaches are becoming more feasible for mass application in humans due to advances in GMP production technologies, production of sporozoites in insect cell lines^26^ and the cryopreservation of sporozoites^27^. Although cross-species protection between *P. cynomolgi* and *P. vivax* remains to be ascertained and while the current approach cannot directly be translated to humans, given the initial success of the hypnoboost concept reported here, approaches using wild-type or engineered *P. cynomolgi* GAP parasites together with selected adjuvants may well be a viable option for the development of a highly efficacious *P. vivax* malaria vaccine.

## Methods

### Ethics statement: study approval and ethics

All non-human primate infections were carried out in accordance with European and Dutch law after positive advice from the ethical committee (DEC). The Council of the Association for Assessment and Accreditation of Laboratory Animal Care (AAALAC International) has awarded BPRC full accreditation.

Non-human primates were used because no other models (in vitro or in vivo) were suitable for the aims of this project. The local independent ethical committee constituted conform Dutch law (BPRC Dier Experimenten Commissie, DEC) approved the research protocol (agreement number DEC# 751B) prior to the start, and the experiments were all performed according to Dutch and European laws. Thus, BPRC is fully compliant with the international demands on animal studies and welfare as set forth by the European Council Directive 2010/63/EU, and Convention ETS 123, including the revised Appendix A as well as the ‘Standard for humane care and use of Laboratory Animals by Foreign institutions’ identification number A5539-01, provided by the Department of Health and Human Services of the United States of America’s National Institutes of Health (NIH) and Dutch implementing legislation. The rhesus monkeys (male Indian *Macaca mulatta*, age 6–15 years) used in this study were captive-bred and socially housed. Animal housing was according to international guidelines for non-human primate care and use. Besides their standard feeding regimen and drinking water ad libitum via an automatic watering system, the animals followed an environmental enrichment programme in which, next to permanent and rotating non-food enrichment, an item of food enrichment was offered to the macaques daily. All animals were monitored daily for health and discomfort. All intravenous injections and large blood collections were performed under ketamine sedation, and all efforts were made to minimize suffering.

### Study design

#### Research objective

Our first objective in this proof-of-concept vaccination experiment is to assess the protective effect of the vaccination protocol, and we expect all animals to be protected by the treatment, while we expect all control animals to become infected. With four animals per group and based on the expected outcome, the following 2 × 2 contingency table (Table 1) can be constructed.

**Table 1:** Statistical power calculation and sample size determination using 2 × 2 contingency table (right) and Fisher’s exact test.

Statistical power	
Observed power (1-β) at *α* = 0.05	0.807
Number required at 1-ß = 0.80 ; *α* = 0.05	8
Number exposed/treated	4
Number controls	4
Required difference |p1-p0|	0.991

Infected			
Vaccinated	Yes	No	Total
Yes	0	4	4
No	4	0	4
Total	4	4	8

#### Sample size

The results were evaluated using Fisher’s exact test, with the data in the contingency table (Table 1) above the Exact P-value (Fisher’s exact test) would be 0.014, with a power of 81% (1-β) to detect a statistically significant difference in treatment effect (development of parasitemia in control versus total protection in the treatment group) and a type I error of 0.05 (α). As relapses cannot be detected under continuous chloroquine coverage and we want to additionally assess the role of blood stages in this model, a further four animals were included for this purpose. This allows making the best use of the additional animals as we both monitor the relapses, which is needed to assess timelines of the vaccination protocol and the role of blood stages through this single group.

#### Animal selection and randomization

A total of twelve healthy male Indian rhesus macaque (*Macaca mulatta*) animals (*N* = 12) were selected, and ten of them were randomized (matching criteria: age and weight) over three groups (*N* = 4 per group; *N* = 2 for the control group): the CPS (hypnoboost-CPS), blood-stage exposed hypnoboost (hypnoboost-BS) and control groups. Two of the twelve animals (R07095, R08100) had previously seen a *P. cynomolgi* sporozoite infection three years before this study and were, therefore, specifically assigned to the control group. All animals were humanely sacrificed at the end of the study.

#### Obtaining sporozoites for infection

In order to obtain *P. cynomolgi* M strain sporozoites for the infection of the hypnoboost-BS groups, *An. stephensi* mosquitoes were fed using membrane feeding on infected blood derived from a *P. cynomolgi*-infected rhesus macaque (donor monkey)^28^. Briefly, rhesus macaques were infected with 1 × 10^6 ^*P. cynomolgi* M strain blood-stage parasites and bled at peak parasitemia. Approximately 300 female *Anopheles stephensi* mosquitoes strain Sind-Kasur Nijmegen were fed with this blood. *An. stephensi* adult mosquitoes were obtained from the Radboud University in Nijmegen. Sporozoites were isolated from the salivary glands of *An. stephensi* mosquitoes and used as detailed below.

#### Experimental design of CPS in the *P. cynomolgi*-*Macaca mulatta* model

After a baseline bleeding (t = 0) was taken to collect plasma and PBMC, the hypnoboost-CPS and hypnoboost-BS groups were infected i.v. with 10^6^ freshly isolated *P. cynomolgi* M strain sporozoites. From day seven post-infection onwards, the hypnoboost-CPS and hypnoboost-BS groups were monitored for parasitemia using thigh prick (thin film smears). Additionally, on day seven post-infection, the hypnoboost and control groups were treated with 7,5 mg/kg i.m. chloroquine (CQ), while, in the blood-stage exposed hypnoboost group, animals were treated with CQ after primary parasitemia was detected starting on day 13 to eliminate the blood stages. At the time of treatment, parasitemia in this group ranged from 0.13 to 2.2% (Supplementary data: table 1). Following the CPS chloroquine administration schedule^3^, hypnoboost and control groups were treated with i.m. chloroquine (CQ) to provide continuous coverage, while treatment of the blood-stage exposed hypnoboost group with i.m. CQ was stopped after primary parasitemia was cured to allow for the monitoring of relapses and exposure to blood stages. To allow for more detailed monitoring of the immune response, on day 14 post-infection (t = 14), another blood sample was taken from all animals (plasma, PBMC).

The blood-stage exposed hypnoboost group was monitored for a total of three relapses before all animals (including the control group) were radically cured by administering 1.8 mg/kg primaquine (PQ) per os in combination with chloroquine (CQ) i.m. for seven days. The animals were trained to drink syrup containing primaquine, which masked the bitter taste of the drug. Compliance was high in all animals; in rare cases in which the animal refused to drink the medication, it was given through gavage. After the radical cure, all animals rested for a period of at least 15 days to allow for the complete washout of residual drug. Thereafter, following a large bleed to collect plasma and PBMC, all animals were challenged with 200 freshly isolated *P. cynomolgi* sporozoites. Starting on day eight after the challenge, the animals were monitored daily for parasitemia through a thigh prick (thin film smears). In terms of relapse, animals were called positive when at least one parasite was seen in slides on two subsequent days.

#### Thin smears and blinding

Thin smears were obtained using a drop of blood, fixed with methanol and stained with Giemsa (Sigma–Aldrich) according to the manufacturer’s specifications. At each time point, at least 20,000 uninfected RBCs were counted to ensure the accuracy of the count. Blood smears to determine parasitemia development after the challenge were read by multiple staff (blinded to the treatment group), to determine the day of first parasitemia in every monkey.

#### Data inclusion and exclusion criteria

No inclusion or exclusion criteria were applied; possible outliers are also shown.

#### Statistical method applied to the evaluation of challenge results

The log-rank test (R survival package) was used to compare the time to parasitemia pair-wise between the three groups, revealing that there is a statistically significant difference between the control and hypnoboost-BS group (*p* = 0.006). Details in Supplementary Fig. 1.

#### Harvesting of plasma

The whole blood of adult rhesus macaques was collected in heparin by saphenous vein puncture under ketamine anaesthesia. Plasma was collected by centrifugation (10 min at 524 × *g*, room temperature (RT)) of the heparin blood using an Allegra XR-15 Centrifuge (Beckman Coulter) and stored at −80 °C.

#### Isolation and concentration of plasma-derived IgGs used in immunofluorescence assay

IgGs were isolated from 1 mL blood plasma collected at four different time points from each of the 12 animals. Twelve Bio-Rad’s Econo-Column Chromatography columns fitted with Bio-Rad’s Econo-Column Flow adaptors were run simultaneously, 1 column per animal. For each animal, the four different time points were run over one column in chronological order. Each column was rinsed with PBS and 0.02% NaN_3_ in between the runs^29^.

#### Antibody determination

The antibody determination was carried out by three independent IgG ELISAs for each antigen (PcyAMA1, PcyMSP1, PcyRON4, PcyRON5, PcyHSP70 or PcyEXP1) in half area microlon 600 microplates (Greiner), each coated overnight at +4 °C with the specific antigen at concentrations of 1 µg/mL (0.05 µg/well). Briefly, after blocking with 100 µl/well 3% BSA/PBST for 2 h at 37 °C, the plasma samples in 0.05% Tween-20/1% BSA/PBS were added in a two-fold dilution series (starting at a dilution of 1:50 for t = 0, at 1:125 for t = challenge and at 1:250 for the plasma harvested at the end of the study) for the IgG ELISAs plasma dilution started at 1:50 for t = 0, at 1:125 for t = 14, t = challenge and at 1:250 for the plasma harvested at the end of the study. The ELISAs were incubated 2 h at 37 °C. As positive control a pool of end sera was used and was used in a two-fold dilution series starting at 1:2000. Antibodies in the plasma samples were detected using the conjugates Rabbit anti-Rat-HRPO (DAKO cytomation cat#P0450) 1:8000 and Goat anti-Hu-IgM-HRP (Southern Biotech #2020-05) 1:2000, 1 h at 37 °C. The ELISA was developed using 50 µl TMB (DIAsource ImmunoAssays S.A. cat# SB04/B), stopped with 50 µl 0.2 M H_2_SO4 (DIAsource ImmunoAssays S.A. cat# SS02-01) and measured on a plate reader (BioRad iMark) at 450 nm. Antibody titres were calculated with Excell and Adamsel, graphs were made in Prism^30^. The different *P. cynomolgi* protein antigens were produced as reported below.

#### Antigen production and purification

Synthetic genes optimized for the indicated microorganisms were purchased from Genscript (Piscataway,NJ).

The gene coding for the ectodomain of PcyAMA1 M-strain (PlasmoDB accession nr. PcyM_PlasmoDB PcyM_0938200, aa 43–487) was expressed in *Pichia pastoris* using the pPicZ alpha A vector and purified using the vector’s encoded hexa-histidine tag^31^.

A gene encoding the C-terminal of PcyMsp1 (PcyMSP1_19_) [PlasmoDB PcyM_0731200, aa1686–1778] was expressed in *Pichia pastoris* using the pPicZ alpha A vector and purified using the vector’s encoded hexa-histidine tag^32^.

The C-terminal of PcyHSP70 [Genbank AAA29625, aa 350–686] was expressed in *E.coli*^33^. An N-terminal hexa-histidine tag was used to purify the protein in a single step using a nickel-activated IMAC (HiTRAP IMAC FF, VWR, Amsterdam, The Netherlands).

The leader sequence-less PcyEXP1 protein, equipped with an N-terminal hexa-histidine tag, [Genbank accession nr XP_004222438, aa 19–133], the N-terminal end of PcyRON4 (PlasmoDB accession nr. PcyM_0918500, aa 1 to 219) and the gene coding for a part of the N-term of the PcyRON5 gene (PcyM_0518100, aa 95–199, Mw 11 kDa) both in frame with the C-terminal vector-encoded hexa-histidine tag were all cloned into the PjExpress412 vector (DNA20/ATUM, Newark, CA), transformed to and expressed in electrocompetent *E. coli* DE3 cells (Thermofisher, Landsmeer, The Netherlands^34^).

#### Immunofluorescence assay (IFA)

IFA were carried out on slides (10-wells slides) coated with *P. cynomolgi* M strain blood stage or sporozoites. Briefly, IFA slides were fixed in cold MeOH for 20 s and air-dried. Of the protG purified monkey IgGs a five-fold dilution series was made, starting at 200 µg/mL in PBS/1%FCS to 1,6 µg/mL. Wells on the IFA slides were incubated with the primary Abs for 1 hr @RT in a moist box. Slides were washed with PBS and incubated with DAPI (D3571; Life Technologies) and Goat-a-Monkey IgG-FITC (ab112766, Abcam; @ 1/1000). Slides were washed, and surface was gently dried. A few drops of Citifluor AF1 Mountant Solution (#17970-25; Electron Microscopy Sciences) were applied before the coverslip was added. IFAs were viewed using the Leica Fluorescence Microscope^35^.

#### Flow cytometric analysis of peripheral blood mononuclear immune cell (PBMC) subsets

PBMCs were profiled by flow cytometry to monitor the frequencies of major subsets (see Supplementary Table 3 for the antibody-conjugate panels used).

Cryopreserved PBMCs were thawed quickly and washed twice in a culture medium with 10% FCS. Cells were counted and brought to a concentration of 0.5 × 10^6^ cells/ml in 0.5% FCS/PBS. Aliquots of 150 µL (1.5 × 10^6^ cells) were seeded in a 96-wells plate, washed with PBS and stained for 20 min at RT with 50 µL Live/Dead™ Fixable Blue dead cell stain (Supplementary Table 3). Thereafter, the cells were washed again with PBS and stained for 30 min at 4 °C with the antibody mix in brilliant-stain buffer (Supplementary Table 3). After staining, all samples were washed twice with 0.5% FCS/PBS and fixed with 2% paraformaldehyde for 1 h before the flow cytometric measurement was performed using an Aurora spectral analyzer (Cytek Biosciences). All analyses were performed using the FlowJo 10.8 software (BD Biosciences). The cytometry gating strategy is specified **in** Supplementary Fig. 2.

### Reporting summary

Further information on research design is available in the Nature Research Reporting Summary linked to this article.

## Supplementary information

Supplementary material one pdf

Supplemtary data

REPORTING SUMMARY

## Data Availability

The materials that support the findings of this study are available from the corresponding author upon request. All data needed to evaluate the conclusions in this paper are present in the paper or the Supplementary Materials.
