# Supplementary material for: Sterile protection against relapsing malaria with a single-shot vaccine
Source: NPJ Vaccines. 2022 Oct 27;7:126. doi: 10.1038/s41541-022-00555-0 (PMC9612615; doi:10.1038/s41541-022-00555-0)
Supplement: Supplementary file 1 — Supplementary material one pdf [file 41541_2022_555_MOESM1_ESM.pdf]

Supplementary Fig. 1. Details of the statistical analysis performed on the challenge data. The log-rank test (R survival package) was used to compare the time to parasitemia pair-wise between the three groups, revealing that there is a statistically significant difference between the control and hypnoboost-BS group ($p=0.006$; log-rank test (R survival package)).

	N	Observed	Expected	(O-E)^2/E	(O-E)^2/V
**Control**	4	4	1,38	4,982	7,842
**Hypnoboost-CPS**	4	3	2,64	0,050	0,081
**Hypnoboost-BS**	4	1	3,98	2,234	5,328

Chisq = 9.6 on 2 degrees of freedom, p = 0.008

	N	Observed	Expected	(O-E)^2/E	(O-E)^2/V
**Hypnoboost-CPS**	4	3	1,46	1,618	2,7
**Hypnoboost-BS**	4	1	2,54	0,932	2,7

Chisq = 2.7 on 1 degrees of freedom, p = 0.1

	N	Observed	Expected	(O-E)^2/E	(O-E)^2/V
**Control**	4	4	1,7	3,11	7,6
**Hypnoboost-BS**	4	1	3,3	1,6	7,6

Chisq = 7.6 on 1 degrees of freedom, p = 0.006

	N	Observed	Expected	(O-E)^2/E	(O-E)^2/V
**Control**	4	4	2,28	1,291	2,67
**Hypnoboost-CPS**	4	3	4,72	0,625	2,67

Chisq = 2.7 on 1 degrees of freedom, p = 0.1

Supplementary Fig. 2. Gating strategy for cytometric analysis of PBMC

Flow cytometry data were cleaned up and major subsets analyzed as illustrated by a representative example and described here in brief. Dead cells were excluded by gating those negative for the dead stain marker [*step 1*] and cell aggregates by gating singlets from FSC-Area versus FSC-Height and a SSC-Area versus SSC-Height dot plots [*steps 2* and *3*]. The time parameter was used to check and exclude any possible anomalies in the acquisition [*step 4*]. Any possible debris was excluded by size gating on a FSC-A:SSC-A plot [*step 5*]. Major lymphocyte subsets were gated as CD20+ B cells or CD3+ T cells [*step 6*], while gamma/delta-TcR-expressing T cells were analysed by separate subgating of the CD3+ T cells [*step 7*]. Non-gamma/delta-T lymphocytes were broken down by gating CD4+ versus CD8+ subsets [*step 8*]. CD3- CD20- non-lymphocytes were analyzed further by gating MHC class II-expressing cells (staining brightly positive with the HLA-DR-specific conjugate) as antigen presenting cells (APC) versus MHC class-II negative innate lymphoid cells of intermediate FSC or low FSC, respectively [*Step 9*]. APC were subdivided by CD14 versus CD16 into CD14 single positives (conventional monocytes, Mo), CD14+CD16+ double positives (intermediate Mo), CD16 single positives and double negatives (DC, or inflammatory Mo), respectively [*step 10*]. CD14-FSC^{im} and CD14-FSC^{low} innate lymphoid cells were segregated further by CD8 vs CD16 expression [*steps 11* and *12*].

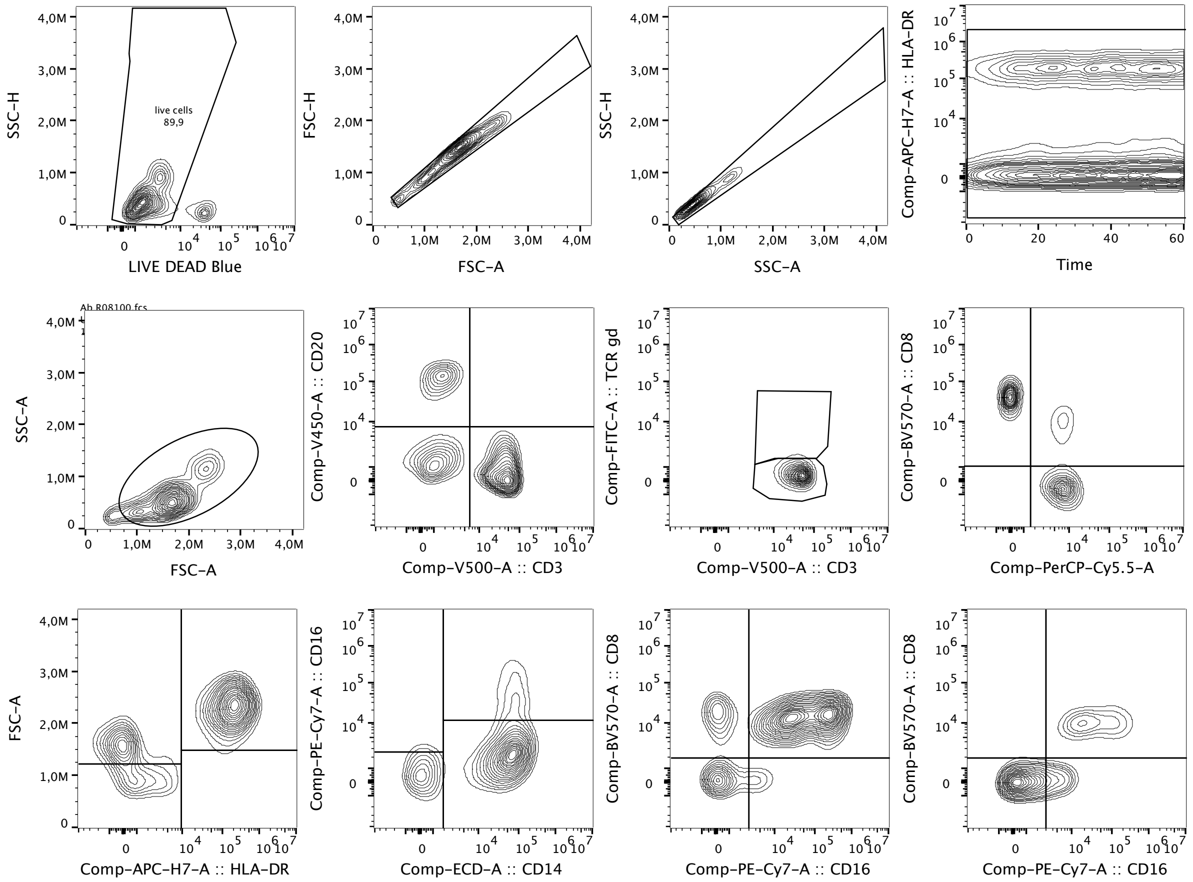

SSC-H
LIVE DEAD Blue
live cells
89,9
FSC-H
FSC-A
SSC-H
SSC-A
Comp-APC-H7-A :: HLA-DR
Time
SSC-A
FSC-A
Comp-V450-A :: CD20
Comp-V500-A :: CD3
Comp-FITC-A :: TCR gd
Comp-V500-A :: CD3
Comp-BV570-A :: CD8
Comp-PerCP-Cy5.5-A
FSC-A
Comp-APC-H7-A :: HLA-DR
Comp-PE-Cy7-A :: CD16
Comp-ECD-A :: CD14
Comp-BV570-A :: CD8
Comp-PE-Cy7-A :: CD16
Comp-BV570-A :: CD8
Comp-PE-Cy7-A :: CD16

Supplementary Table 3 – Overview of the antibody panel used in the flowcytometric analysis of peripheral blood mononuclear immune cell subsets

antibody-conjugate / reagent	brand	dilution	clone	Cat#	Lot#
Live/Dead™ Fixable Blue Dead Cell Stain	Invitrogen	1:4000	n.a.	L23105	2214471
HLA-DR - APC-H7	BD Biosciences	1:80	L243	641411	7205849
Pan TCR-gd - FITC	BioLegend	1:40	B1	331208	B226477
CD20 - V450	BD Bioscences	1:80	L27	561163	2356914
CD3 - V500	BD Bioscences	1:40	SP34-2	560770	1060836
CD4 - PerCP-Cy5.5	BD Bioscences	1:200	L200	552838	6175960
CD8a - BV570	BioLegend	1:80	RPA-T8	301037	B173337
CD14 -ECD	Beckman Coulter	1:50	RM052	B92391	63
CD16 - PE-Cy7	BD Biosciences	1:80	3G8	557744	6237704
