# Supplementary material for: Sterile protection against relapsing malaria with a single-shot vaccine
Source: NPJ Vaccines. 2022 Oct 27;7:126. doi: 10.1038/s41541-022-00555-0 (PMC9612615; doi:10.1038/s41541-022-00555-0)
Supplement: Supplementary file 3 — REPORTING SUMMARY [file 41541_2022_555_MOESM3_ESM.pdf]

nature portfolio

Corresponding author(s): Clemens H. M. Kocken

Last updated by author(s): Jul 6, 2022

Reporting Summary

Nature Portfolio wishes to improve the reproducibility of the work that we publish. This form provides structure for consistency and transparency in reporting. For further information on Nature Portfolio policies, see our Editorial Policies and the Editorial Policy Checklist.

Statistics

For all statistical analyses, confirm that the following items are present in the figure legend, table legend, main text, or Methods section.

n/a	Confirmed	
☐	☒	The exact sample size (*n*) for each experimental group/condition, given as a discrete number and unit of measurement
☒	☐	A statement on whether measurements were taken from distinct samples or whether the same sample was measured repeatedly
☐	☒	The statistical test(s) used AND whether they are one- or two-sided<br>*Only common tests should be described solely by name; describe more complex techniques in the Methods section.*
☒	☐	A description of all covariates tested
☒	☐	A description of any assumptions or corrections, such as tests of normality and adjustment for multiple comparisons
☐	☒	A full description of the statistical parameters including central tendency (e.g. means) or other basic estimates (e.g. regression coefficient) AND variation (e.g. standard deviation) or associated estimates of uncertainty (e.g. confidence intervals)
☒	☐	For null hypothesis testing, the test statistic (e.g. *F*, *t*, *r*) with confidence intervals, effect sizes, degrees of freedom and *P* value noted<br>*Give P values as exact values whenever suitable.*
☒	☐	For Bayesian analysis, information on the choice of priors and Markov chain Monte Carlo settings
☒	☐	For hierarchical and complex designs, identification of the appropriate level for tests and full reporting of outcomes
☒	☐	Estimates of effect sizes (e.g. Cohen's *d*, Pearson's *r*), indicating how they were calculated

Our web collection on statistics for biologists contains articles on many of the points above.

Software and code

Policy information about availability of computer code

Data collection	N/A
Data analysis	N/A

For manuscripts utilizing custom algorithms or software that are central to the research but not yet described in published literature, software must be made available to editors and reviewers. We strongly encourage code deposition in a community repository (e.g. GitHub). See the Nature Portfolio guidelines for submitting code & software for further information.

Data

Policy information about availability of data

All manuscripts must include a data availability statement. This statement should provide the following information, where applicable:

- Accession codes, unique identifiers, or web links for publicly available datasets
- A description of any restrictions on data availability
- For clinical datasets or third party data, please ensure that the statement adheres to our policy

The materials that support the findings of this study are available from the corresponding author upon request. All data needed to evaluate the conclusions in this paper are present in the paper or the Supplementary Materials.

Human research participants

Policy information about studies involving human research participants and Sex and Gender in Research.

Reporting on sex and gender	N/A
Population characteristics	N/A
Recruitment	N/A
Ethics oversight	N/A

Note that full information on the approval of the study protocol must also be provided in the manuscript.

Field-specific reporting

Please select the one below that is the best fit for your research. If you are not sure, read the appropriate sections before making your selection.

☒ Life sciences ☐ Behavioural & social sciences ☐ Ecological, evolutionary & environmental sciences

For a reference copy of the document with all sections, see nature.com/documents/nr-reporting-summary-flat.pdf

Life sciences study design

All studies must disclose on these points even when the disclosure is negative.

Sample size

Our first objective in this proof of concept vaccination experiment is to see the protective effect of the vaccination protocol and we expect all animals to be protected by the treatment, while we expect all control animals to become infected. With four animals per group and based on the expected outcome, the following 2 x 2 contingency table can be constructed.
Observed Power (1-ß) at alpha = 0.05 0.807
Number required at 1-ß = 0.80 ; alpha = 0.05 => 8 animals
Number exposed/treated: 4
Number controls: 4
Required Difference |p1-p0| 0.991
A 2x2 contingency table was used and results were evaluated using Fisher's exact test, with the data in the contingency table above the Exact P-value would be 0.014, with a power of 81% (1-ß) to detect a statistically significant difference in treatment effect (development of parasitemia in control versus total protection in treatment group) and a type I error of 0.05 (alpha).

Data exclusions

None

Replication

Describe the measures taken to verify the reproducibility of the experimental findings. If all attempts at replication were successful, confirm this OR if there are any findings that were not replicated or cannot be reproduced, note this and describe why.

Randomization

A total of twelve healthy male Indian rhesus macaque (Macaca mulatta) animals (N=12) were selected and randomized (matching criteria: age and weight) over three groups (N=4 per group): the CPS (hypnoboost), blood stage exposed hypnoboost and control groups. Two animals (R07095, R08100) assigned to the control group had previously seen a sporozoite infection in 2014.

Blinding

Blood smears to determine parasitemia development after challenge were read by multiple staff (blinded to the treatment group), to determine day of first parasitemia in every monkey.

Reporting for specific materials, systems and methods

We require information from authors about some types of materials, experimental systems and methods used in many studies. Here, indicate whether each material, system or method listed is relevant to your study. If you are not sure if a list item applies to your research, read the appropriate section before selecting a response.

Materials & experimental systems

n/a	Involved in the study
☐	☒ Antibodies
☐	☒ Eukaryotic cell lines
☒	☐ Palaeontology and archaeology
☐	☒ Animals and other organisms
☒	☐ Clinical data
☒	☐ Dual use research of concern

Methods

n/a	Involved in the study
☒	☐ ChIP-seq
☐	☒ Flow cytometry
☒	☐ MRI-based neuroimaging

Antibodies

Antibodies used

Antibody: HLA-DR - APC-H7 Brand: BD Biosciences clone: L243 Cat#: 641411 Lot#: 7205849
Antibody: Pan TCR-gd - FITC Brand: BioLegend clone: B1 3Cat#: 31208 Lot#: B226477
Antibody: CD20 - V450 Brand: BD Bioscences clone: L27 Cat#: 561163 Lot#: 2356914
Antibody: CD3 - V500 Brand: BD Bioscences clone: SP34-2 Cat#: 560770 Lot#: 1060836
Antibody: CD4 - PerCP-Cy5.5 Brand: BD Bioscences Cat#: 552838 Lot#: 6175960
Antibody: CD8a - BV570 Brand: BioLegend clone: RPA-T8 Cat#: 301037 Lot#: B173337
Antibody: CD14 -ECD Brand: Beckman Coulter Cat#: PNIM2707U Lot#: 63
Antibody: CD16 - PE-Cy7 Brand: BD Biosciences clone: 3G8 Cat#: 557744 Lot#: 6237704

Validation

All monoclonals have been validated for the their cross reactivity with non-human primates.

Eukaryotic cell lines

Policy information about cell lines and Sex and Gender in Research

Cell line source(s)

Pichia pastoris Km71H

Authentication

Cell line was purchased at ThermoFisher, and not further authenticated.

Mycoplasma contamination

Not tested for Mycoplasma

Commonly misidentified lines
(See ICLAC register)

Pichia pastoris has been renamed “Komagataella phaffii”

Animals and other research organisms

Policy information about studies involving animals; ARRIVE guidelines recommended for reporting animal research, and Sex and Gender in Research

Laboratory animals

Male Indian Macaca mulatta infected with P. cynomolgi M strain

Wild animals

N/A

Reporting on sex

All animals were male

Field-collected samples

N/A

Ethics oversight

BPRC Dier Experimenten Commissie, DEC approved the research protocol (agreement number DEC# 751B)

Note that full information on the approval of the study protocol must also be provided in the manuscript.

Flow Cytometry

Plots

Confirm that:

☒ The axis labels state the marker and fluorochrome used (e.g. CD4-FITC).

☒ The axis scales are clearly visible. Include numbers along axes only for bottom left plot of group (a 'group' is an analysis of identical markers).

☒ All plots are contour plots with outliers or pseudocolor plots.

☒ A numerical value for number of cells or percentage (with statistics) is provided.

Methodology

Sample preparation	Cryopreserved PBMCs were thawed quickly and washed twice in media + 10% FCS. Cells were counted and brought to a concentration of 0.5*106 cells/ml in 0,5% FCS/PBS. 150 µL (1.5*106) cells were aliquoted in a 96-wells plate, washed with PBS and stained for 20 min at RT with 50 µL live dead blue, fluorescent reactive dye (Invitrogen Cat#L23105A lot#2214471;1:2000). Thereafter, the cells were washed again with PBS and stained for 30 min at 4□C with the antibody mix in brilliant-stain buffer (Supplementary Table 3). After staining, all samples were washed twice with 0.5% FCS/PBS and fixed with 2% paraformaldehyde for 1 hour before the flowcytometric measurement was performed .
Instrument	The flowcytometric measurement was performed using an Aurora spectral analyzer (Cytek Biosciences)
Software	All analyses were performed using the FlowJo 10.7 software (BD Biosciences).
Cell population abundance	No sorting was performed
Gating strategy	A representative gating strategy is depicted in Supplementary Figure 2

☒ Tick this box to confirm that a figure exemplifying the gating strategy is provided in the Supplementary Information.
